# Do plants use root-derived proteases to promote the uptake of soil organic nitrogen?

**DOI:** 10.1007/s11104-020-04719-6

**Published:** 2020-09-23

**Authors:** Lucy M. Greenfield, Paul W. Hill, Eric Paterson, Elizabeth M. Baggs, Davey L. Jones

**Affiliations:** 1 School of Natural Sciences, Bangor University, Gwynedd, LL57 2UW UK; 2 The James Hutton Institute, Craigiebuckler, Aberdeen, AB15 8QH UK; 3 Global Academy of Agriculture and Food Security, the Royal (Dick) School of Veterinary Studies, University of Edinburgh, Easter Bush Campus, Midlothian, EH25 9RG UK; 4 SoilsWest, UWA School of Agriculture and Environment, The University of Western Australia, Perth, WA 6009 Australia

**Keywords:** Aminopeptidase, Peptidase, Plant nutrition, Proteinase, Root exudation

## Abstract

**Aims:**

The capacity of plant roots to directly acquire organic nitrogen (N) in the form of oligopeptides and amino acids from soil is well established. However, plants have poor access to protein, the central reservoir of soil organic N. Our question is: do plants actively secrete proteases to enhance the breakdown of soil protein or are they functionally reliant on soil microorganisms to undertake this role?

**Methods:**

Growing maize and wheat under sterile hydroponic conditions with and without inorganic N, we measured protease activity on the root surface (root-bound proteases) or exogenously in the solution (free proteases). We compared root protease activities to the rhizosphere microbial community to estimate the ecological significance of root-derived proteases.

**Results:**

We found little evidence for the secretion of free proteases, with almost all protease activity associated with the root surface. Root protease activity was not stimulated under N deficiency. Our findings suggest that cereal roots contribute one-fifth of rhizosphere protease activity.

**Conclusions:**

Our results indicate that plant N uptake is only functionally significant when soil protein is in direct contact with root surfaces. The lack of protease upregulation under N deficiency suggests that root protease activity is unrelated to enhanced soil N capture.

**Electronic supplementary material:**

The online version of this article (10.1007/s11104-020-04719-6) contains supplementary material, which is available to authorized users.

## Introduction

The rhizosphere represents a zone of intense competition for nutrient resources between plant roots and soil microorganisms (Jones et al. 2009). This competition is particularly intense for low molecular weight forms of organic N such as amino acids, oligopeptides and urea which can be taken up and assimilated by both plants and microorganisms (Kuzyakov and Xu 2013; Moreau et al. 2019). Conventionally, it is thought that high molecular weight N held in soil organic matter is largely unavailable to plants and that this resource needs to be hydrolysed to induce solubilisation and promote plant availability (Schulten and Schnitzer 1997). This hydrolysis step has been shown to be a major bottleneck in N cycling in many ecosystems (Jan et al. 2009). Of the organic N held in soil organic matter, ca. 40% is typically present in the form of protein which enters soil mainly from plant and microbial necromass. Microorganisms release extracellular protease and deaminase enzymes into the soil to break down this protein into oligopeptides, amino acids and NH_4_^+^. The soluble products can then a) be taken up and assimilated by the microbial community and any excess NH_4_^+^ excreted back into the soil, or b) taken up directly by plant roots and associated mycorrhizas (Schimel and Bennett 2004). However, some studies have reported that plant roots can also release extracellular proteases into the soil (Adamczyk et al. 2010). Although plant roots contain a wide range of intracellular proteases (Tornkvist et al. 2019), the production of extracellular proteases by plant roots has been hypothesised to have at least four distinct functions: 1) enhancing availability of N for nutrition, 2) defence against plant pathogenic organisms, 3) root cell expansion, and 4) regulation of proteins and peptides in response to developmental and environmental cues (i.e. signal transduction; van der Hoorn 2008; Kohli et al. 2012). In addition, roots may unwittingly release proteases into soil during apoptotic cell death (e.g. from border cells or epidermal and cortical cell death) or following lysis caused by mesofaunal damage or physical abrasion (e.g. root hairs) (Wen et al. 2007; Sun et al. 2015; Song et al. 2016). Theoretically, the use of root proteases to promote organic N release may reduce competition with microorganisms, given that only a small proportion of the root surface is colonised by microorganisms (Foster 1986). In addition, it may allow the spatially targeted release of exoenzymes at sites where the N demand is greatest (e.g. root tips). This would be similar to the well-established mechanism of phosphatase release from roots experiencing P limitation (Ciereszko et al. 2011).

Evidence that plant root proteases can increase the supply of N from the soil remains conflicting. For example, Godlewski and Adamczyk (2007) report that 15 different agricultural and wild plant species have the ability to release proteases. Also, their studies on *Triticum aestivum* (Adamczyk et al. 2008) and *Allium porrum* (Adamczyk et al. 2009; Adamczyk 2014) indicate that these proteases may increases levels of free amino acids in the soil. Paungfoo-Lonhienne et al. (2008) have also observed that plants can secrete root proteases but that they also have the potential to take up exogenously supplied proteins intact via endocytosis. In contrast, Chang and Bandurski (1964) and Vágnerová and Macura (1974) both reported negligible root protease activity in cereals, while Eick and Stöhr (2009) showed no change in membrane-bound protease activity under N deficient conditions. Similarly, Synková et al. (2016) and Paungfoo-Lonhienne et al. (2008) have shown that *Nicotiana tabacum*, *Hakea actites* and *Arabidopsis thaliana* plants grow very poorly when supplied just with protein. Lastly, an upregulation of protease activity may occur under different nutritional stresses (e.g. P deficiency) suggesting that the response is not N-specific (Tran and Plaxton 2008). These differences in opinion could be attributed to the different methods used to measure protease activity and plant growth conditions (German et al. 2011). This is particularly the case when sampling the root secretome due to (i) the release of intracellular proteases from roots damaged during handling, (ii) contamination from seed exudates known to be rich in proteases, (iii) and difficulties in achieving or maintaining sterile conditions, particularly the elimination of root endophytes (Sánchez-López et al. 2018; Oburger and Jones 2018).

This study focuses on aminopeptidases (E.C.3.4.11) which catalyse the cleavage of *N*-terminus amino acids from peptide and protein substrates. They are involved in fundamental plant cellular processes (e.g. mitosis, meiosis, oxidative regulation) and in various aspects of plant development via degradation of storage protein (e.g. germination, senescence) (Oszywa et al. 2013; Kania and Gillner 2015; Budic et al. 2016). Plants typically encode many aminopeptidases (e.g. *Arabidopsis thaliana* encodes at least 28) which can have broad specificity (Ogiwara et al. 2005; Walling 2006). Scranton et al. (2012) found that leucine aminopeptidase can moonlight as a molecular chaperone to aid plant defence. In addition, aminopeptidases are induced under both drought and metal stress in the plant roots (Wang et al. 2011; Boulila-Zoghlami et al. 2011). Importantly, aminopeptidases have also been implicated in autophagy under N deficiency (Xia et al. 2012; Xu et al. 2019), suggesting that they are a good candidate to investigate for their role in protein-N mobilisation a rhizosphere context.

Investigations of the role of plant proteases in N acquisition have generally focused on proteases secreted from roots (Vágnerová and Macura 1974; Godlewski and Adamczyk 2007). Proteomic studies of the apoplast and cell wall, however, have revealed the presence of a wide range of proteases, most of which have unknown roles (Rodríguez-Celma et al. 2016; Calderan-Rodrigues et al. 2019). Therefore, with a focus on aminopeptidases, our aim was to determine: a) whether plants release free proteases from their roots or if the proteases remain root surface-bound, b) if proteins and/or their breakdown products are taken up by the plant, c) if root protease activity is up- or down-regulated in the presence of inorganic N and, d) how root protease activity compares to rhizosphere protease activity. We hypothesise that plants will both secrete proteases from their roots but also retain surface-bound protease activity to maximise protein-N capture from soils. We also expect protease activity to be induced in the absence of an inorganic N supply (Godlewski and Adamczyk 2007). Finally, we hypothesise that protease activity from rhizosphere soil will be proportionally higher than for roots as it is more energetically favourable for the soil microbial community to use the products of protein hydrolysis rather than inorganic N (Abaas et al. 2012)*.*

## Materials and methods

### Growth of plants

Maize (*Zea mays* L.) and wheat (*Triticum aestivum* L.) were chosen as the study species as both plants are cereals with wide agricultural use but have different N use efficiencies (Liang et al. 2013). Seeds were surface sterilised by shaking with 70% ethanol for 5 min and then with 10% sodium hypochlorite containing one drop of Tween 20 for 5 min. The seeds were then rinsed four times in sterile, deionised water. The seeds were germinated and grown for up to two weeks in sterile Phytatrays® (Sigma-Aldrich, Poole, UK) on autoclaved agar with either inorganic N or zero N nutrient solution added. Seedlings were grown at 20 °C, 12 h photoperiod at 500 μmol photons m^−2^ s^−1^ PAR.

### Nutrient solution

Seedlings were supplied with either a zero N nutrient solution or inorganic N nutrient solution in the agar. The zero N nutrient solution consisted of 1.5 mM MgSO_4_, 2 mM K_2_SO_4_, 4 mM CaCl_2_, 1.87 mM NaH_2_PO_4_, 0.13 mM Na_2_HPO_4_, 0.14 mM H_3_BO_3_, 0.02 mM MnSO_4_, 0.002 mM ZnSO_4_, 0.003 mM CuSO_4_, 0.0002 mM Na_2_MoO_4_, 0.089 mM Fe(III)-citrate in 0.1 mM of MES buffer (pH 5.6) (Hewitt 1966). The inorganic N solution consisted of 4 mM NaNO_3_ and 4 mM NH_4_Cl in addition to the zero N nutrient solution.

### Extracellular root protease: Proteases in solution

After one-week, sterile seedlings (*n =* 8 for each treatment per plant) of similar height and root length were transferred from the Phytatrays® into a pre-autoclaved hydroponic growth system. The plants were firstly placed into a 1.5 ml Eppendorf tube with the bottom removed. This was then placed into the top of a 50 cm^3^ polypropylene centrifuge tube containing nutrient solution and then into a larger sterile box. Nutrient solution was injected into each centrifuge tube via silicone tubing connected to a 0.22-μm filter located outside the box. The nutrient solution in the centrifuge tube was continually aerated by passing 0.22-μm filtered air into the solution via silicone tubing located outside the box. An air outlet from the centrifuge tube was via silicon tubing with a hydrophobic 0.22-μm filter (Supporting information, Fig. S1). Weekly, nutrient solutions were removed from the hydroponic system through a 0.22-μm filter and protease activity measured. Fresh nutrient solution was then injected into each centrifuge tube through a 0.22-μm filter. Nutrient solutions were changed weekly to ensure nutrients were never limited and provide a weekly time series of protease activity over the seedlings growth. A negative control consisted of nutrient solution with no plant present. All work was carried out in a sterile, laminar flow cabinet. After four weeks of growth, under the constant conditions outlined previously, the experiment was stopped. The roots and shoots were separated, the fresh weight recorded, then oven dried at 80 °C for 24 h after which the dry weight was recorded.

To ensure that the system was sterile, an open Petri-dish with nutrient agar was placed at the bottom of the hydroponic system. At the end of the experiment, nutrient solution was plated onto nutrient agar. If no microbial growth was observed after one week at 37 °C, the system was considered sterile.

### Protease assay

Leucine aminopeptidase activity was used as an exemplar to measure potential protease activity according to Vepsäläinen et al. (2001). The nutrient solution was pipetted (100 μl) into a 96 well plate. Substrate (100 μl of 500 μM L-leucine 7-amido-4-methylcoumarin hydrochloride dissolved in sterile water and passed through a 0.22-μm filter to ensure no microbial contamination) was added to the sample (pH 5.7). Standards were prepared for each sample by adding 100 μl of 7-amido-4-methylcoumarin (7-AMC) at different concentrations (0, 0.5, 1, 5, 10, 15, 25 and 50 μM) to 100 μl of sample for quench correction. After a 3 h incubation at 20 °C, fluorescence was measured at an excitation wavelength of 335 nm and emission wavelength 460 nm on a Cary Eclipse Fluorescence Spectrophotometer (Agilent Corp., Santa Clara, CA). A calibration curve was then fitted for each sample. Blank sample and substrate measurements were subtracted from the assay reading.

### Extracellular root protease: Proteases in the root

To determine surface bound root protease activity, we carried out a protease assay in situ. After two weeks of growth, plants (*n* = 4) were transferred into a sterile 50 cm^3^ centrifuge tube where the protease assay was carried out as described above except the assay solution consisted of 5 ml of sterile nutrient solution and 5 ml of 500 μM L-leucine 7-amido-4-methlycoumarin hydrochloride. Plants were incubated at 20 °C for 3 h in the sterile laminar flow cabinet. The plants were removed and 200 μl of assay solution were pipetted into a 96-well plate for fluorescence measurement. At the end of each experiment, roots and shoots were separated and the fresh weight recorded, then oven dried at 80 °C for 24 h and the dry weight recorded (Supporting information, Fig. S2).

### ^14^C-protein uptake experiment

To determine whether plants use protein and/or its derivatives as a sole N source we carried out a ^14^C-protein uptake experiment. After two weeks of growth, plants (*n =* 4) were removed from the nutrient agar and placed in 10 mL sterile zero N nutrient solution in a 50 cm^3^ sterile centrifuge tube in a laminar flow cabinet. Each plant was placed in a sterile plastic air-tight box. Uniformly ^14^C-labelled protein from *Nicotiana tabacum* L. leaves (1 ml; 0.064 mg C l^−1^; 0.0063 mg N l^−1^; 3.3 kBq ml^−1^; >3 kDa; custom produced by American Radiolabeled Chemicals, St Louis, MO) was secondary purified by ultrafiltration in an Amicon® stirred cell using a 3 kDa Ultracel® cutoff membrane (Millipore UK Ltd., Watford, UK) to remove any oligopeptides and pipetted into the nutrient solution. To capture the ^14^CO_2_ evolved from plant respiration a 1 M NaOH trap (1 ml) was added to the box. After 24 h the plants were removed, and the roots washed in 0.1 M CaCl_2_. The roots and shoot were separated, weighed and dried at 80 °C for 24 h. To measure the ^14^C in the root and shoot biomass, the dried samples were oxidised on a Harvey OX400 Biological Oxidiser (Harvey Instruments Corp., Hillsdale, NJ, USA) and ^14^CO_2_ captured in Oxysolve C-400 Scintillant (Zinsser Analytic, Frankfurt, Germany) and ^14^C determination using a Wallac 1414 scintillation counter with automated quench correction (PerkinElmer Inc., Waltham, MA). The amount of ^14^CO_2_ captured was determined after addition of Optiphase HiSafe3 scintillation fluid to the NaOH traps and ^14^C determination using a Wallac 1414 scintillation counter with automated quench correction (PerkinElmer Inc.). We acknowledge that we do not know the forms of ^14^C that were taken up into the plant (i.e. intact protein or hydrolysis products such as peptides or amino acids), but we assume it is as an organic N compound.

### Rhizosphere protease activity

To compare root protease activity to rhizosphere soil protease activity, we collected an agricultural topsoil (0–15 cm) from Abergwyngregyn, UK (53°14′29”N, 4°01′15”W). The soil was characterised as a Eutric Cambisol (pH 6.8; 27.8 g C kg; 3.4 g N kg). Soil was sieved (<2 mm) and added to boxes (8 cm × 10.5 cm × 4 cm) to achieve a dry bulk density of 1 g cm^−3^. Maize and wheat seeds were germinated and densely planted in the soil (1 seed per 1 cm^3^) to maximise the rhizosphere effect and grown at 20 °C, 12 h photoperiod at 500 μmol photons m^−2^ s^−1^ PAR. Seedlings were watered daily. After 2 weeks, the rooting was dense and, therefore, all soil was considered to be rhizosphere soil. Soil was sampled and a soil slurry created by adding 0.2 g to 20 ml sterile, 0.1 mM MES buffer (pH 5.6) and shaking for 30 min at 250 rev min^−1^. Protease activity was also measured at the native soil pH (6.8) in a soil slurry with sterile, deionised water (1:100 soil:water ratio). Protease activity did not significantly differ between the two assay pHs (unpaired t test: *p* = 0.21). Rhizosphere protease activity was compared to extracellular root protease activity under inorganic N treatment for each species. We determined the volume of root to be 0.00785 cm^3^ for maize and 0.00502 cm^3^ for wheat with 1 cm root length and 1 mm and 0.8 mm diameter for maize and wheat, respectively (Eq. 1).

1 $$ \mathrm{Volume}\ \mathrm{of}\ \mathrm{root}\ \left({cm}^3\right)=\uppi {r}^2h $$

We assumed the root density to be 1 g cm^−3^ and, thus, the fresh root weight to be 0.00785 g and 0.00502 g for maize and wheat respectively. Assuming, 90% water, the dry root weight is 0.000785 cm^3^ and 0.000502 g cm^−3^ (Eq. 2).

2 $$ Dry\  roo t\ weight=0.1\frac{1\ g\ {cm}^{-3}}{Volume\ of\  roo\mathrm{t}\ \left({cm}^3\right)} $$

We determined the rhizosphere extent to be 2 mm from the root surface. Therefore, the volume of soil surrounding 1 cm of root would be 0.126 cm^3^ (Eq. 1). The soil dry bulk density is 1 g cm^−3^, thus, the soil weight would be 0.126 g. We then determined the final soil weight surrounded by the root to be 0.118 g and 0.121 g of soil for maize and wheat respectively (Eq. 3).

3 $$ Final\ soil\ weight\ (g)= total\ soil\ weight\ (g)- dry\  weight\ of\ root\ (g) $$

Rhizosphere protease activity was then compared to extracellular root protease activity (μmol AMC cm^−1^ root h^−1^).

### Statistical analysis

All experiments were performed in quadruplicate. All statistical analyses were performed on R version 3.5.0 (R Core Team 2018). Normality of the data was determined by Shapiro-Wilk test (*p* > 0.05) then visually checked using qqnorm plots. Homogeneity of variance of the data was determined by Bartlett test (*p* > 0.05) then visually checked using residuals vs. fitted plots. One-way ANOVAs were used to determine if there was a significant difference (*p* < 0.05) between N treatment for extracellular protease activity and ^14^C-labelled protein uptake for each species. Unpaired t-tests were used to determine if there was a significant difference (*p* < 0.05) between rhizosphere and extracellular root protease activity.

## Results

### Root protease activity

**Fig. 1:**
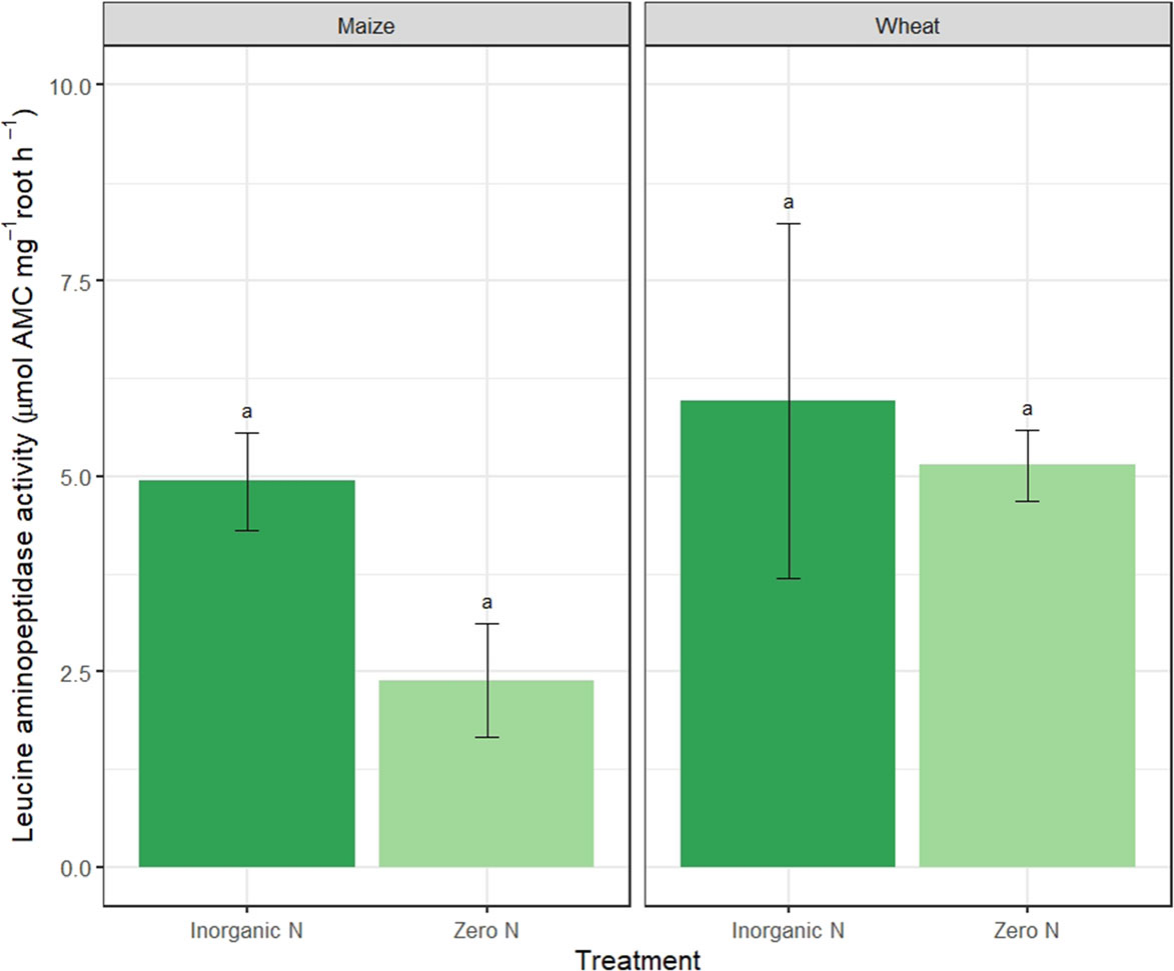
Extracellular root leucine aminopeptidase activity (μmol AMC mg^−1^ root h^−1^ of maize and wheat under inorganic N and zero N treatments measured using the in situ assay. Different letters represent significant difference between N treatments for each plant (*p* < 0.05). Values represent mean ± SEM (*n* = 4)

We found no evidence of protease activity in the nutrient solution that the seedlings were grown in (no significant difference from the control, unpaired t-test: *p* = 0.84; data not presented). However, we did observe measurable protease activity in the in-situ protease assay. Extracellular root protease activity ranged from 2 to 5 μmol AMC mg^−1^ root h^−1^ in maize roots and 5–6 μmol AMC mg^−1^ root h^−1^ in wheat roots (Fig. 1). We assume all protease activity measured in situ to be extracellular root protease at or in the root surface because we found no evidence when protease activity was measured in the solution only. Protease activity was not significantly different between N treatments, but under the N-addition treatments, protease activity was two times higher for maize and ca. 14% higher for wheat (F_(1,6) =_ 6.4, *p* = 0.53 and F_(1,6) =_ 0.13, *p* = 0.73, respectively).

### ^14^C-protein uptake

**Fig. 2:**
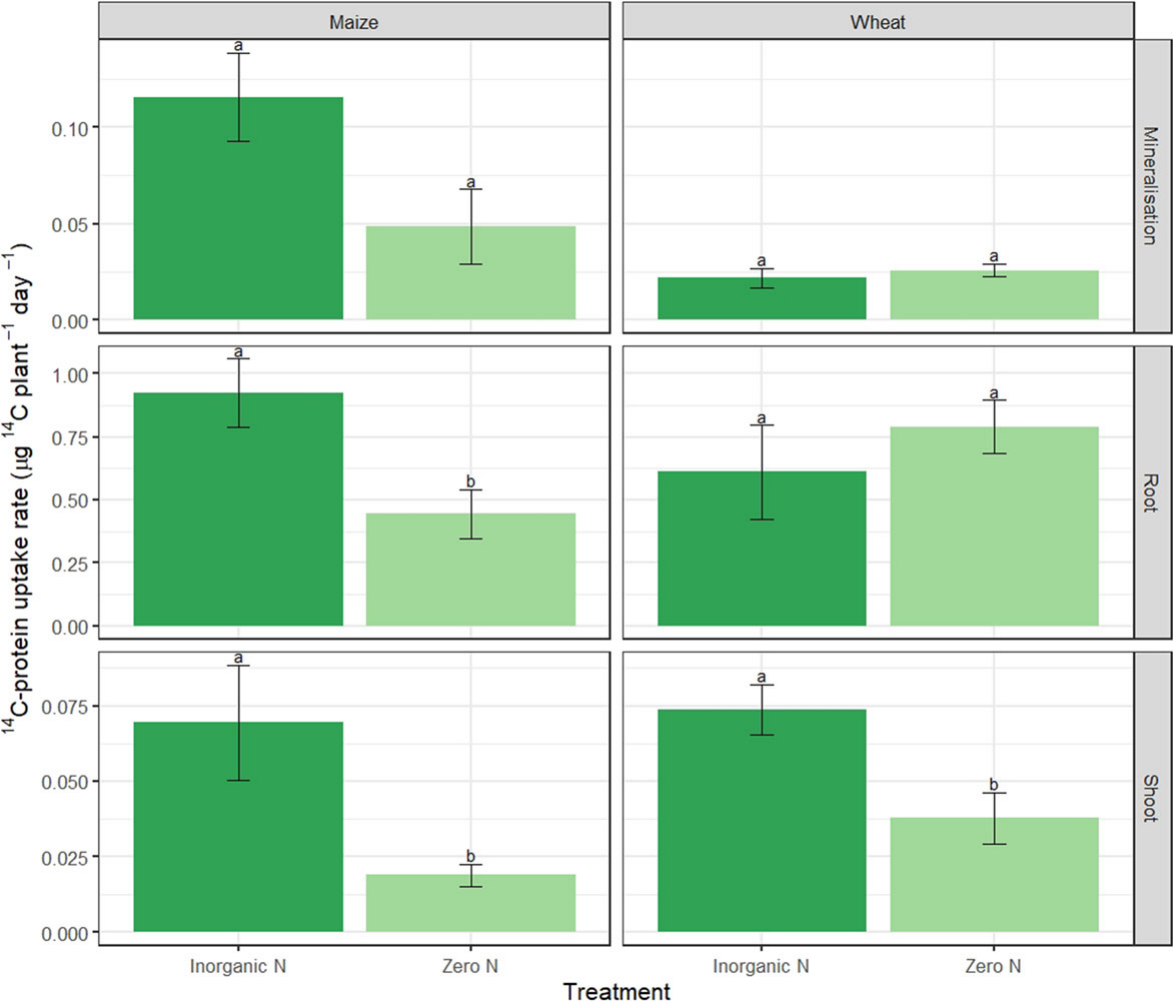
^14^C-labelled protein respired, root and shoot uptake rate (μg ^14^C plant^−1^ day^−1^) of maize and wheat under inorganic N and zero N treatments. Different letters represent significant difference between N treatments for each plant (*p* < 0.05). Values represent mean ± SEM (*n* = 4)

We measured plant uptake of ^14^C derived from labelled protein to determine whether the breakdown products from proteolysis were utilised by the plant. Mineralisation of ^14^C-protein to ^14^CO_2_ was similar between N treatments for both maize and wheat (*p* = 0.06 and 0.54 respectively) (Fig. 2). Root uptake of ^14^C was ca. twice as high under the inorganic N than zero N treatment in maize (*p* = 0.03) (Fig. 2). However, wheat root uptake of ^14^C-protein was similar between treatments (*p* = 0.43). Uptake of ^14^C-protein into the plant shoot was ca. three times higher under inorganic N than zero N for maize and ca. twice as high for wheat (*p* = 0.04 and 0.02 respectively) (Fig. 2).

### Rhizosphere and root protease activity

**Fig. 3:**
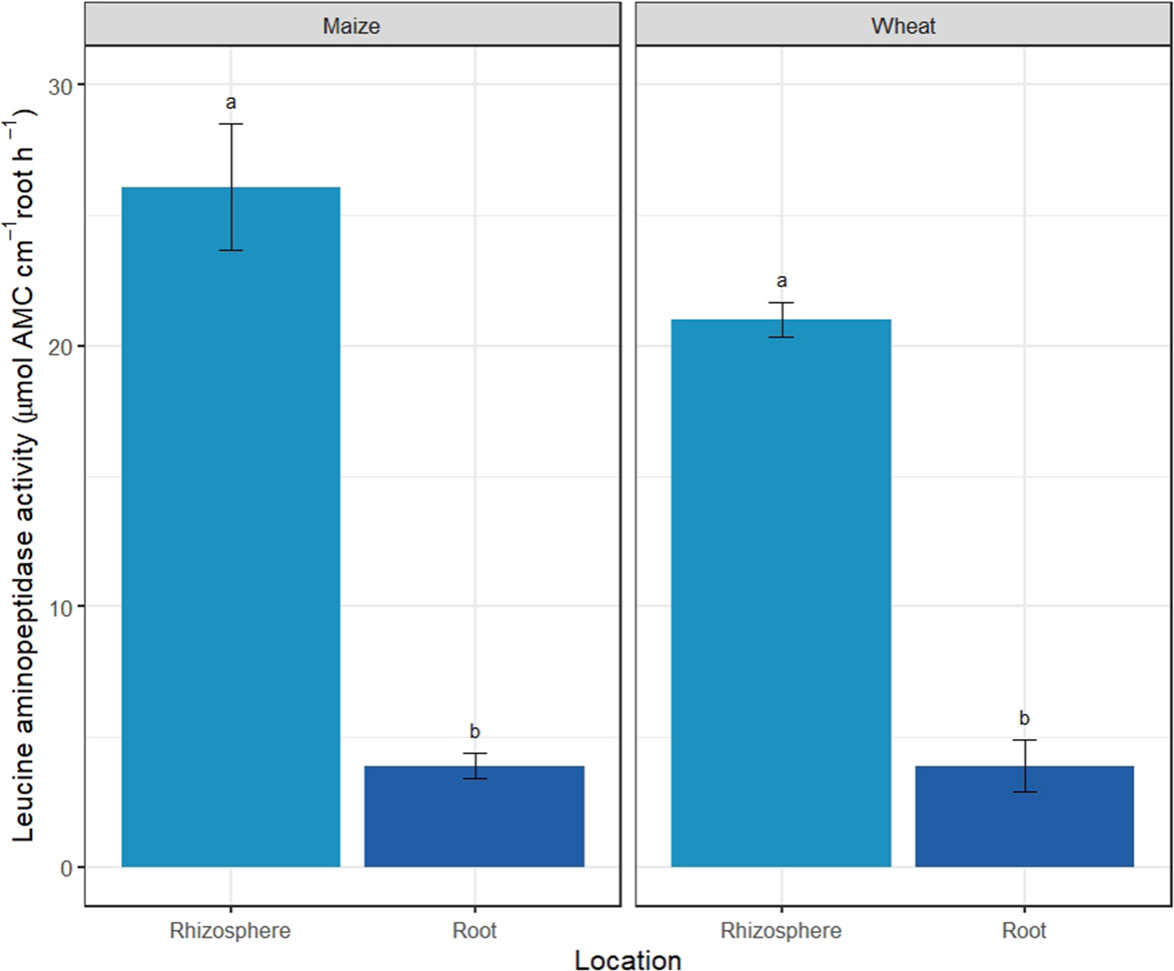
Comparison of leucine aminopeptidase activity in the rhizosphere and extracellular root (μmol AMC cm^−1^ root h^−1^) of maize and wheat. Different letters represent significant difference between N treatments for each plant (*p* < 0.05). Values represent mean ± SEM (*n* = 4)

We compared root protease activity to rhizosphere protease activity to determine the potential ecological significance of plant root protease activity. Extracellular root protease activity contributed 15% and 19% of rhizosphere protease activity (Fig. 3) (t-test: *p* = 0.006 and *p* < 0.0001 for maize and wheat respectively).

## Discussion

### Free versus surface bound root protease activity

**Fig. 4:**
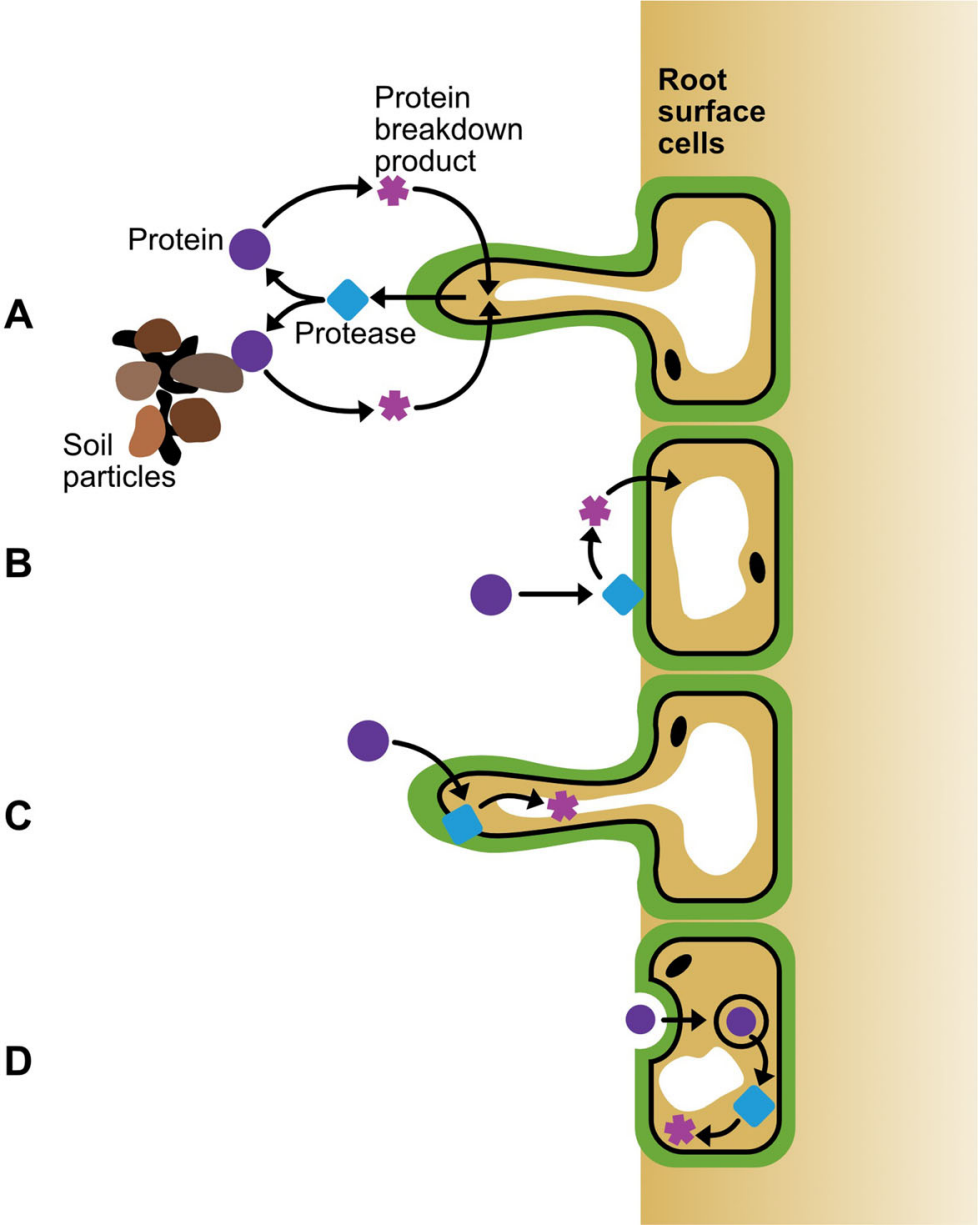
Schematic diagram for the mechanisms of root protease activity in order to obtain N for nutrition

Here we evaluated the possible importance of four different mechanisms for the use of protein-derived N by plant roots, and their likely importance in plant N nutrition: A) Proteases are released from the root into the external medium where they diffuse away and encounter proteins on soil surfaces and/or free in solution and the products released diffuse back to the root where they can be taken up (Adamczyk et al. 2010); B) Proteins come in direct contact with the root surface enabling cleavage by outward facing cell wall bound proteases and uptake of soluble products; C) Proteins diffuse through pores in the cell wall, entering the apoplast where plasma membrane or inward-facing cell wall bound proteases break them into soluble products (Chang and Bandurski 1964); and D) Small proteins are taken up by the root cell via endocytosis (Carpita et al. 1979) (Fig. 4). In this study we found no evidence to show that root proteases are released into the external medium in significant quantities (mechanism A), however, we did find strong evidence for root-bound protease activity (mechanisms B and C). In this study, it was not possible to determine the direct contribution of mechanism D as this can only be confirmed when mechanisms A and B are absent using our methods. Our findings are therefore consistent with studies of plant proteomes which have revealed a high diversity and proportion of proteases among cell wall proteins (ca. 15% of the total; Albene et al. 2014; Canut et al. 2016). These proteases have been shown to be important regulators of plant growth and development, however, their potential role in N nutrition remains unclear (van der Hoorn 2008). Their known functions include: (i) breakdown of cell wall proteins to facilitate cell wall re-organisation (e.g. at the root-symbiont interface), (ii) removal of oxidised/damaged proteins (Takeda et al. 2009), (iii) the production of active peptides important for plant defence responses (immune signalling; Plattner and Verkhratsky 2015; Hou et al. 2018), (iv) the synthesis of anti-microbial peptides (Schaller et al. 2018), (iv) regulators of programmed cell death (phytaspases; Chichkova et al. 2010), (v) cell wall loosening to enable mucilage release (Rautengarten et al. 2008), and (vi) potential salvage of C and N resources in senescing tissues (Polge et al. 2009). To date, all the evidence suggests that these events are highly spatially and temporally co-ordinated in response to specific environmental stimuli and developmental cues (van der Hoorn 2008; Plattner and Verkhratsky 2015). The activity of these proteases also appears to target specific protein substrates, consistent with the view that they are not generalist proteases involved in the breakdown of soil-derived protein. Although there is a lack of evidence for their direct involvement in N nutrition, it is clear that many could have an indirect role on N nutrition; for example, through improved N recycling and N use efficiency in the plant, reducing microbial growth and competition for exogenous N, enhancing soil-root contact, and promoting symbioses that promote N acquisition (e.g. N fixation, mycorrhizas). Of critical significance is that many of these proteases are upregulated in response to environmental stress (e.g. Jorda and Vera 2000; Golldack et al. 2003), a feature that was not seen in our experiments when N was withheld from the plants. This suggests that the degradation of exogenous proteins at the root surface is either a constitutively expressed trait, or more likely is just an indirect consequence of foreign proteins adhering to the root surface or entering the apoplast where proteolysis occurs. A similar argument has been made for the indirect capture of amino acids and peptides from soil as transporters for these solutes are also not up-regulated in cereals under N deficiency (Jones and Darrah 1994). In this latter situation, the active uptake of amino acids and oligopeptides at the epidermal surface and apoplast is likely associated with the recapture of solutes lost in root exudation by passive diffusion (Jones et al. 2009) and not uptake of organic N from soil (Kuzyakov and Xu 2013).

While cell wall proteases may indirectly lead to some cleavage of proteins, further action of cell wall endo/exopeptidases may still be required to transform larger peptides into oligopeptides capable of active transport into the cell. To date, there is no evidence suggesting these enzymes are regulated by plant N status with most implicated in the recycling of damaged proteins (e.g. TPP(II) cell wall exopeptidase; Book et al. 2005; Polge et al. 2009). Again, this indicates that while the root possesses a full complement of enzymatic machinery required for proteolysis and the uptake of soluble products, this may have no direct involvement in N acquisition. One caveat we note is that our study only focused on fluorescent substrates targeted at aminopeptidases. Further studies are warranted on other types of fluorescent substrates which can target alterative proteases.

### Are root proteases quantitatively important in nitrogen uptake from soil?

Most studies on the direct uptake of exogenous proteins by roots have been undertaken in the absence of soil and at very high soluble protein concentrations, conditions that might be viewed as ecologically unrealistic (White et al. 2015). In addition, even when purified protein forms are used these do not represent soil proteins and can still contain substantial amounts of oligopeptide impurities. In our study, we secondary-purified our plant-derived protein to remove oligopeptides, however, this was still added directly to the nutrient medium. In these situations, proteins tend to be attracted to the charged root surface where clumping can occur (White et al. 2015). In soil, however, it is more likely that soluble proteins will preferentially sorb to soil particles and/or denature and precipitate, hampering their movement and bioavailability (Fiorito et al. 2008). This implies that soil-borne protein needs to be in close proximity to the root surface for root-mediated, protein-derived N uptake to occur. This is consistent with our results and others showing that roots contain both inward and outward facing cell wall proteases (Figueiredo et al. 2018; Hou et al. 2018), indicating that they can cleave large proteins outside the cell wall (mechanism B) and either cleave or directly take up smaller ones diffusing through the cell wall (mechanism C and D; Fig. 4).

The ^14^C-labelled proteins used in this study contained a range of molecular weights (3–100 kDa) and therefore sizes. It is likely that this also affects their potential for uptake. Conventionally, the cell wall rather than the plasma membrane is thought to represent the main barrier to protein uptake. This is due to the charged nature of the wall which induces protein binding and retention (Albene et al. 2014), but also due to the small pores (4–5 nm diameter) in the wall which prevents the inward movement of larger proteins (>30 kDa; Palocci et al. 2017). This is consistent with the inward movement and intact uptake of the highly stable, green fluorescent protein (~27 kDa) from solution by *Arabidopsis* roots (mechanism D; Paungfoo-Lonhienne et al. 2008). However, Read and Bacic (1996) suggest that, albeit less frequent, 6–9 nm diameter pores may also exist, which would allow the ingress and potential uptake of much larger proteins (65–100 kDa), although the significance of this pathway remains unknown. We hypothesize that at least some of our ^14^C-labelled proteins would have been capable of passing through the cell wall and being available for root uptake. Unfortunately, the molecular weight distribution of proteins in soil solution remains virtually unknown. Based on the root uptake of a wide range of synthetic nanoparticles (up to 50 nm diameter) it also implies that this is not a protein specific pathway (Lv et al. 2019). Consequently, although evidence exists for low molecular weight protein uptake, it may not necessarily mean that it is quantitatively important in N nutrition.

A study, that investigated whether *Arabidopsis* could use protein as a N source, found that growth was higher in plants grown on a combination of organic and inorganic N sources rather than protein alone (protein and inorganic N > inorganic N > protein) (Paungfoo-Lonhienne et al. 2008). It is therefore possible that plant N limitation could inhibit protease synthesis. However, we would also expect that if outward facing protease activity was a preferred plant strategy under N limitation that it would preferentially allocate N resources to this function. By analogy, in the case of root C starvation, it is well established that a large proportion of intracellular protein can be degraded to provide C skeletons for respiration without a loss of basic metabolism (Brouquisse et al. 1991). It is also possible that the presence of proteins in the rhizosphere could induce extracellular protease production which the absence of proteins in our experiments would have prevented. However, this mechanism has only been observed in fungi so far (e.g. Hanson and Marzluf 1975; Boer and Peralta 2000). In addition, when ^14^C-labelled protein was added, the uptake of ^14^C-derived from protein into the shoot was also higher under the inorganic N treatment. This suggests that proteases are not induced under N deficiency. We hypothesise that the supply of inorganic N drives faster growth which in turn leads to greater cell wall reorganisation, more plasma membrane vesicle fusion events (facilitating protein internalisation) and greater cell wall protease activity.

### Root versus rhizosphere protease activity

Rhizosphere protease activity was higher than extracellular root protease activity for both maize and wheat. We expected rhizosphere protease activity to be high because the rhizosphere is a hotspot for microbial activity (Kuzyakov and Blagodatskaya 2015). Soil microorganisms are largely C limited and they produce proteases to liberate both C and N from proteinaceous compounds, with a large proportion of the protein-C subsequently used in catabolic processes (Gonzales and Robert-Baudouy 1996; Jan et al. 2009). Furthermore, they do not favour the uptake of NO_3_^−^ as this is energetically unfavourable (Abaas et al. 2012). This contrasts with crop plants who often favour NO_3_^−^ as a source of N due to its fast diffusion in soil and who are rarely C limited (Iqbal et al. 2020). Previous reports for protease and other enzymes (e.g. Badalucco et al. 1996; Gramss et al. 1999; Brzostek et al. 2013) have shown that roots contribute little to overall rhizosphere hydrolytic activity. In contrast, our study shows up to one-fifth of rhizosphere protease activity is of root origin. In future, it is important to consider the potential contribution of plant root proteases in rhizosphere activity.

## Conclusions

Although plants have the potential to contribute to rhizosphere protease activity and possess the capacity to take up and metabolise protein breakdown products, current evidence suggests that this plays a minor role in N nutrition. Our study found no evidence for the root-release of proteases into the soil solution. In contrast, we present strong evidence for root-bound protease activity and breakdown of soluble proteins. However, our results suggest that the use of exogenous protein may be an indirect by-product of other processes occurring in the root. In particular, the lack of up-regulation in protease activity under N deficiency and low intrinsic rates of protease activity in comparison to soil microbial-derived protease activity suggests it plays a minor role in overall plant N acquisition.

## Electronic supplementary material

ESM 1 (DOCX 159 kb)

## Data Availability

The data that support the findings of this study are available from the corresponding author upon reasonable request.
