# Supplementary material for: Do plants use root-derived proteases to promote the uptake of soil organic nitrogen?
Source: Plant Soil. 2020 Sep 23;456(1):355–67. doi: 10.1007/s11104-020-04719-6 (PMC7567722; doi:10.1007/s11104-020-04719-6)
Supplement: Supplementary file 1 — (DOCX 159 kb) [file 11104_2020_4719_MOESM1_ESM.docx]

**Plant and Soil Supporting Information**

**Do plants use root-derived proteases to promote the uptake of soil organic nitrogen?**

Lucy M. Greenfield^1*^, Paul W. Hill^1^, Eric Paterson^2^, Elizabeth M. Baggs^3^, Davey L. Jones^1,4^

^1^*School of Natural Sciences, Bangor University, Gwynedd, LL57 2UW, UK*

^2^*The James Hutton Institute, Craigiebuckler, Aberdeen, AB15 8QH, UK*

^3^*Global Academy of Agriculture and Food Security, the Royal (Dick) School of Veterinary Studies, University of Edinburgh, Easter Bush Campus, Midlothian, EH25 9RG, UK*

^4^*SoilsWest, UWA School of Agriculture and Environment, The University of Western Australia, Perth, WA 6009, Australia*

Corresponding author: Lucy M. Greenfield*

Corresponding author address: School of Environment, Natural Resources and Geography,
Bangor University, Bangor, Gwynedd, LL57 2UW, UK

Corresponding author Tel: +447928770387

Corresponding author E-mail: l.greenfield@bangor.ac.uk


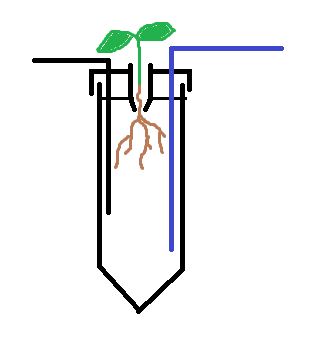


Air supply

Nutrient solution

0.22 µm filter

0.22 µm filter

50 ml centrifuge tube

Nutrient solution

Airtight container

Eppendorf

Air outlet

0.22 µm filter

**Figure S1.** Diagram of the sterile hydroponics setup used for the experiments.


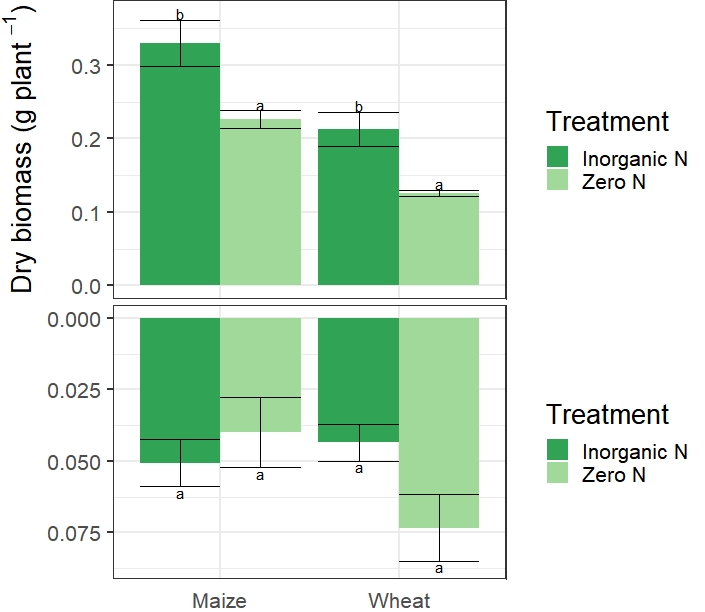


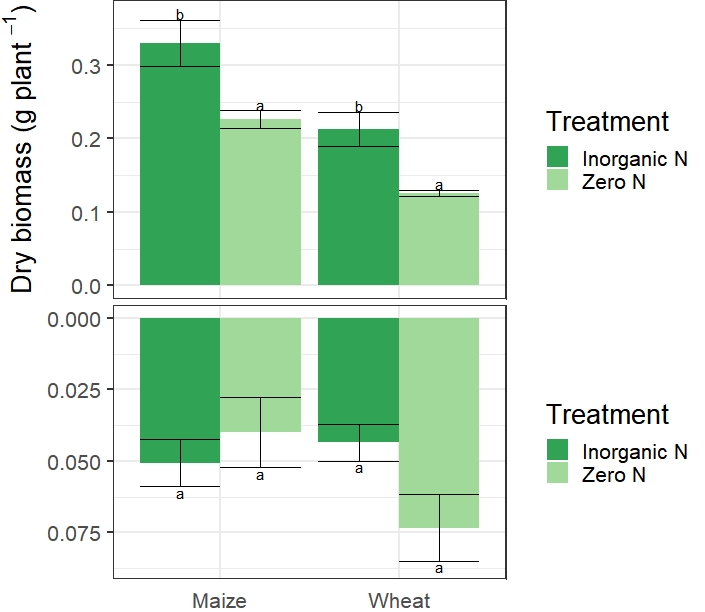


**Figure S2.** Shoot (upper graph) and root (lower graph) dry biomass of maize and wheat under different N treatments after two weeks of growth. Different letters represent significant difference between N treatments for each plant (*p* < 0.05). Values represent mean ± SE (*n* = 4).
